# Developing a Reliable Mouse Model for Cancer Therapy-Induced Cardiovascular Toxicity in Cancer Patients and Survivors

**DOI:** 10.3389/fcvm.2018.00026

**Published:** 2018-04-05

**Authors:** Kyung Ae Ko, Yin Wang, Sivareddy Kotla, Yuka Fujii, Hang Thi Vu, Bhanu P. Venkatesulu, Tamlyn N. Thomas, Jan L. Medina, Young Jin Gi, Megumi Hada, Jane Grande-Allen, Zarana S. Patel, Sarah A. Milgrom, Sunil Krishnan, Keigi Fujiwara, Jun-Ichi Abe

**Affiliations:** ^1^Department of Cardiology, The University of Texas MD Anderson Cancer Center, Houston, TX, United States; ^2^Department of Radiology Oncology, The University of Texas MD Anderson Cancer Center, Houston, TX, United States; ^3^Texas A&M Chancellor Research Initiative, Prairie View A&M University, Prairie View, TX, United States; ^4^Department of Bioengineering, Rice University, Houston, TX, United States; ^5^KBRwyle, Science and Space, Technology and Engineering, Houston, TX, United States

**Keywords:** Cancer treatment-related cardiovascular toxicity, atherosclerosis, disturbed blood flow, p90RSK, ionizing radiation

## Abstract

**Background:**

The high incidence of cardiovascular events in cancer survivors has long been noted, but the mechanistic insights of cardiovascular toxicity of cancer treatments, especially for vessel diseases, remain unclear. It is well known that atherosclerotic plaque formation begins in the area exposed to disturbed blood flow, but the relationship between cancer therapy and disturbed flow in regulating plaque formation has not been well studied. Therefore, we had two goals for this study; (1) Generate an affordable, reliable, and reproducible mouse model to recapitulate the cancer therapy-induced cardiovascular events in cancer survivors, and (2) Establish a mouse model to investigate the interplay between disturbed flow and various cancer therapies in the process of atherosclerotic plaque formation.

**Methods and Results:**

We examined the effects of two cancer drugs and ionizing radiation (IR) on disturbed blood flow-induced plaque formation using a mouse carotid artery partial ligation (PCL) model of atherosclerosis. We found that doxorubicin and cisplatin, which are commonly used anti-cancer drugs, had no effect on plaque formation in partially ligated carotid arteries. Similarly, PCL-induced plaque formation was not affected in mice that received IR (2 Gy) and PCL surgery performed one week later. In contrast, when PCL surgery was performed 26 days after IR treatment, not only the atherosclerotic plaque formation but also the necrotic core formation was significantly enhanced. Lastly, we found a significant increase in p90RSK phosphorylation in the plaques from the IR-treated group compared to those from the non-IR treated group.

**Conclusions:**

Our results demonstrate that IR not only increases atherosclerotic events but also vulnerable plaque formation. These increases were a somewhat delayed effect of IR as they were observed in mice with PCL surgery performed 26 days, but not 10 days, after IR exposure. A proper animal model must be developed to study how to minimize the cardiovascular toxicity due to cancer treatment.

## Introduction

Cancer therapy, including anthracyclines, cisplatin, tyrosine kinase inhibitors, hormone deprivation agents, and ionizing radiation (IR) can affect not only the heart but also the vasculature and its reactivity. For example, Chaosuwannakit et al. showed that proximal aortic wall stiffness increased 3 months after anti-cancer treatments after they had standardized other potential contributing factors such as age, sex, diabetes, and hypertension. The authors showed that the aortic stiffness increase occurred soon after the administration of cancer therapy and was equivalent to the stiffness increase due to 10 to 20 years of aging (1, 2). Of note, aortic stiffness increases with age (3), cardio-metabolic abnormalities (4, 5), and increased sodium intake (6), all of which are associated with left ventricular dysfunction (LVD) (7). The accelerated aortic stiffness in the patients with Hutchinson-Gilford progeria syndrome (premature aging) is well known (8). Importantly, the strong association between arterial stiffness and increased ventricular afterload, LVD, hypertrophy, and adverse vascular events including atherosclerosis and subsequent CV mortality has been well established (9, 10). In fact, higher incidence of CV events in cancer survivors has also been well established.

Recently, possible involvement of vascular dysfunction in the initiation of cancer therapy-related cardiac dysfunction (CTRCD) has been suggested. For example, Narayan et al. reported that ventricular-arterial coupling (effective arterial elastance [Ea]/end-systolic elastance [Ees_sb_]) could be used to predict CTRCD (11). Importantly, they found that the association between Ea/Ees_sb_ and CTRCD was driven primarily by Ea. Therefore, they suggested that increased arterial elastance and alterations in arterial stiffness in ventricular-arterial coupling occur with and in advance of ejection fraction** (**EF) deterioration. Furthermore, Drafts et al. reported that thoracic aortic pulse wave velocity (PWV), as detected by phase-contrast cardiac magnetic resonance, increased within 6 months after a low to moderate dose of anthracycline-based cancer therapy, suggesting that cancer therapy is associated with an early and persistent increase in aortic PWV, which is a measure of arterial/aortic stiffness (12). Interestingly, Ohyama et al. recently reported a strong correlation between aortic arch stiffness and LV function (13). They found that a higher aortic arch PWV was associated with impaired LV systolic function. The association between PWV and a lower early diastolic strain rate, a marker of early diastolic dysfunction, was also demonstrated in this MRI study (13). Taken together, these data suggest that cancer therapy can affect not only cardiac function but also vascular function and subsequent development of atherosclerosis.

Radiation therapy (RT) is one of the crucial interventions to control primary thoracic malignancies, breast cancer, and lymphoma. It is now clear, however, that RT causes delayed negative effects on the CV system, leading to morbidity and mortality among cancer survivors. For example, among the survivors of Hodgkin’s lymphoma, CV events after RT to the chest area are increased by up to 7-fold based on multivariate analyses controlling for other risk factors (14), and CV events including ischemic heart disease and heart failure are the leading causes of non-oncologic deaths (15). In addition, significantly increased mortality due to CV disease in breast cancer survivors after RT has also been reported(16). In patients with locally advanced non-small cell lung cancer, the 2 year incidence of grade ≥3 CV toxicity induced by RT was 11% in a combined analysis of 4 prospective studies (17). Lastly, it has been suggested that deaths linked to unavoidable IR exposure of the heart could offset any benefit of increasing the radiation dose to control the disease (18). Therefore, RT-induced CV toxicity is a significant factor when determining the survival of cancer patients who receive RT to the upper body. The incidence of numerous cardiovascular disorders is increased after RT, including coronary artery disease, congestive heart failure, and valvular disorders (19).

It is interesting to note that various forms of cancer treatments whose mechanisms of action are different cause similar cardiovascular pathological phenotypes. For example, Lipshultz et al. have reported that cancer survivors treated by so-called “non-cardiotoxic” drugs show pathological cardiovascular phenotypes. They have shown that not only the survivors treated with cardiotoxic drugs, but also those treated with so-called “non-cardiotoxic” drugs also exhibit decreased LV mass and cardiac dysfunction compared to healthy siblings. In addition, cancer survivors exposed to both cardiotoxic and so-called “non-cardiotoxic” drugs had a higher mean body mass index, higher fasting serum non-high-density-lipoprotein, higher insulin levels, and higher sensitivity C-reactive protein levels, suggesting that all cancer survivors exposed to any type of cancer therapy had a high risk of CTRCD (20). Lipshutz’s group suggested that one of the common phenotypes observed in these patients is premature aging (20).

There are two types of blood flow in large arteries, namely, laminar flow (l-flow) and disturbed flow (d-flow). It has been well established that atherosclerotic plaques are rare in areas exposed to laminar flow, which induces anti-inflammatory signaling and anti-atherogenic gene expression in endothelial cells (ECs) (21, 22). In contrast, atherosclerotic plaques localize to areas of d-flow found in regions where vessels curve acutely, bifurcate, or branch. Previously, we have reported that d-flow induces p90RSK activation and expression (23), and up-regulates inflammation, apoptosis, and proliferation of ECs; and reduces vascular reactivity (24). Since both IR and d-flow can increase EC activation (25), it is important to study the possible interplay between d-flow and cancer treatments in regulating EC functions, but to our knowledge, there has been no study focused on the simultaneous assault of these two stimuli on ECs. Therefore, we decided to establish an animal model to investigate the effect of various cancer treatments including IR on d-flow-induced plaque formation in this study. The goals of this study are: (1) to generate an affordable, reliable, and reproducible mouse model that recapitulate the cancer therapy-induced cardiovascular events in cancer survivors and (2) to establish a mouse model to investigate the interplay between disturbed flow and various cancer therapies in the process of atherosclerotic plaque formation.

## Methods

Antibodies utilized for immunohistochemistry and immunofluorescence were against smooth muscle actin (SMA;#M0851, DAKO, CA), MAC3 (#550292, BD, NJ), pan p90RSK (cat #MAB2056, Novus Biologicals, LLC, CO), and phospho-p90RSK (Ser380, # 9341) from Cell Signaling Technology (Danvers, MA).

### Doxorubicin and Cisplatin Treatment

Mice received doxorubicin (5 mg/kg of body weight; intraperitoneal injection; Cat# NDC 67457-436-50, Mylan Institutional LLC, IL) or saline on day 0, 7 and 14.

Mice received cisplatin (2.3 mg/kg of body weight; intraperitoneal injection; Cat# NDC 0703-5747-11, Teva Pharmaceuticals USA, Inc., PA) for 5 consecutive days, rested for the next 5 days, and then restarted the same daily injections for 5 more days. In the preliminary experiments, we found that mice showed severe weight loss after the cisplatin treatment. Therefore, we add 0.5 ml of phosphate-buffered solution (PBS; NaCl 137 mmol/L, KCl 2.7 mmol/L, Na_2_HPO_4_ 10 mmpl/L, KH_2_PO_4_ 1.8 mmol/L) subcutaneous infusion immediately after cisplatin treatment. The treatment regime we described in this study is within the range of human treatment regime variation (26–28). As we describe repeatedly the purposes of this study are (1) to generate tan affordable, reliable, and reproducible mouse model to recapitulate the cancer therapy-induced cardiovascular events in cancer survivors, and (2) to generate a mouse model to investigate the interplay between disturbed flow and various cancer therapy in the process of atherosclerotic plaque formation. And our purpose is not to find the regimen to cure cancer. Therefore, the rationales we used for these treatment regimens are: (1) the treatment regimen is within the range of human treatment and also used in the previous studies in mice and (2) the treatment regimens should not significantly affect the health of each mouse.

### Housing and Husbandry

Mice were housed in pathogen-free conditions at the Texas A&M Institute of Biosciences and Technology and the University of Texas MD Anderson Cancer Center. The Program for Animal Resources is AAALAC certified and defined pathogen free facility for housing mice and rats. Cage level barrier system was used, diet was irradiated, ultra-filtered water, heat treated wood chip bedding. And enrichment material (nestlets) was provided. Cages and water were changed on a regular weekly basis. Animals were handled under hepa-filtered change station. Environmental parameters (room lighting, temperature and humidity) were computer monitored as follows, (1) Temperature: Set point 72F (high limit 74F, low limit 70F), (2) Humidity: Set point 45% (high limit 55%, low limit 40%), (3) Light cycle: 12 h light, 12 h dark, (4) Air changes 10–15 times per hour. The vivarium was staffed seven days a week by animal caretakers, including week-ends & holidays. Veterinary care and oversight was provided by contract veterinarian who visited facilities on a regular basis and was also available for consultation by phone/email.

### Mice, Left Carotid Artery Partial Ligation, and Atherosclerosis

*Ldlr*^−/−^ mice were obtained from The Jackson Laboratory, Bar Harbor, ME, USA. Eight- to twelve-week-old mice were fed an adjusted-calorie (high-fat) diet (HFD) consisting of 21% crude fat, 0.15% cholesterol, and 19.5% casein (cat. no. TD.88137; Envigo, NJ, USA) (24) as indicated. Since we did not find a significant difference in the response to cancer therapy, we used both males and females. The number of each sex in each experiment was added in each figure. The weight range of the mice was 15–25 g. Genotyping was performed based on the Jackson Lab protocol, and we confirmed that the mice were homozygous. No previous procedures were performed before the experiments in this study. To induce atherosclerosis by d-flow, we performed partial ligation of the left carotid artery (LCA) as we had described (23). We conducted a double-blind, randomized study and the persons who evaluated the size of the plaque were blinded until the data analysis was complete. Briefly, mice were anesthetized by 2.0% isoflurane, placed on a heated surgical pad, and subcutaneously given 5mg/kg Caprofen. Isoflurane was maintained at a level between 1.0 and 2.0%. Classic Vaporizer unit from Braintree Scientific (Cat. # EZ-150C) was used for the delivery and regulation of isoflurane during surgery. We followed the approved protocol by IAUCC and IBT committee for the choice of anesthetic drug, route of administration, and dose. After hair removal, a midline cervical incision was made and the internal and external carotid arteries were exposed and partially ligated with 6.0 silk suture, leaving the occipital artery patent (Figure S1). The skin was sutured with absorbable 6.0 silk suture in a running subcuticular pattern. Mice were allowed to recover in a clean cage on a heated pad. For the atherosclerosis study, mice were fed a HFD (TD88137, Envigo), at which time their carotid arteries were harvested. Each experiment was performed during 9 AM to 5 PM at central standard time in USA. Each experiment was performed in the vivarium designated for animal surgery at UT MD Anderson Cancer Center and at the Texas A&M Institute of Biosciences and Technology, Houston TX, 77030. Experimental procedures were approved by the Institutional Animal Care and Use Committee (IACUC) at Texas A&M Institute of Biosciences and Technology and also at the University of Texas MD Anderson Cancer Center.

### Monitoring of Mice After Surgery

Mice were monitored until recovery in a chamber on a heating pad following surgery. We injected postoperative analgesia as needed for additional pain relief. We supported the post-operated mice by (1) Warmth: they were placed in a pre-warmed cage (cage was on a heating pad), (2) Fluids: pre-warmed normal saline (37**°**C**,** 5 ml per 100 g body weight) was given subcutaneously. We checked on mice every 10 min until mice were awake and moving. Daily for the first 3 days (date of surgery was day 1), then once per week afterwards till conclusion of study as described in our protocol approved by the IAUCC and IBT committee.

### Sample Size Calculation

Initially we performed the sample size calculation based on our previous results (23). We expected that (1) the variation (SD) of lesion size in each group will be around 30% from the mean value, (2) we can detect 100% increase in the lesion size by cancer therapy, and (3) the variation within the vehicle control and the cancer therapy will be similar. Therefore, the optimal number of animals needed to attain statistical significance of *p* < 0.05 with a 90% probability is 5 (based on the power calculator generated by the Laboratory Animal Services Centre at The Chinese University of Hong Kong: http://www.lasec.cuhk.edu.hk/sample-size-calculation.html).

### Histology and Evaluation of Atherosclerotic Lesions After Partial Carotid Ligation

To determine disturbed flow-induced atherosclerotic lesions in histological sections, right (control) and left (surgery performed) carotid arteries were dissected out and all tissues were fixed in 10% neutralized buffered formalin. The fixed tissues were embedded in paraffin. Serial sections (5 µm) were made through the entire carotid arteries and stained with Masson’s trichrome or H&E (Figure S1). To quantify atherosclerotic lesions, the intima area was calculated by subtracting the lumen area from the area circumscribed by the internal elastic lamina. The medial area was determined by the area between the internal and the external elastic laminae. These measurements were made by using ImageJ (http://imagej.nih.gov/ij/). The positions of the internal and the external elastic laminae were also confirmed by Masson’s trichrome staining. The extent of atherosclerotic lesion was determined by detecting the intimal and media layers in both left and right carotid arteries.

### Immuno-Histochemistry

To identify macrophages and smooth muscle cells (SMCs) within the plaque area, immunohistochemistry (IHC) was performed. Epitope retrieval (HIER) was performed by heating de-paraffinized slides in the HIER buffer containing 10 mM sodium citrate and 0.05% Tween 20 (pH 6) at 100**°**C for 10–15 min. After cooling the slides down to room temperature (RT), slides were treated with 3% hydrogen peroxide and then were blocked with 5% normal goat serum (Vector laboratories) for 30 min at RT. Primary antibodies were against Mac-3 (1:100, Rat, BD550292) for macrophages and α-smooth muscle actin (SMA) (1:500, Mouse, Ab7817) for SMCs. Secondary antibodies (goat anti-mouse or anti-rat-Biotinylated) were used at 1:1,000 dilutions. Sections were developed by DAB substrate (ImmPACT DAB, SK-4105) and counterstained with hematoxylin.

### Grading of Necrotic Core Formation

To quantify necrotic core formation, cross-sectioned carotid arteries were stained by hematoxylin and eosin, and the necrotic core formation was quantified by % of non-cellular area/total lesion area by using ImageJ (http://imagej.nih.gov/ij/). We graded each necrotic core as no necrotic core = 0, ≤5% = 1 or >5% =2 and scored at seven different levels within each carotid artery after partial carotid ligation as shown in Figure S1. For each mouse, the sum of the total grades was calculated.

### Immunofluorescence Staining

Immunofluorescence staining was performed on paraffin slides as described previously (29). Briefly, the tissue sections were de-paraffinized and incubated with 10% normal goat serum for 30 min. Epitope retrieval (HIER) was performed by boiling de-paraffinized slides in the HIER buffer containing 10 mM sodium citrate and 0.05% Tween 20 (pH 6) at 100**°**C for 20 min. The slides were then incubated with primary antibodies at 4°C overnight, followed by incubation with Alexa Fluor 647-conjugated secondary antibodies for 60 min at RT. Expression of total p90RSK and phospho-p90RSK (S380) were imaged on an Olympus FX1200 confocal laser scanning microscope.

### Irradiation

Mice were irradiated on a Cesium-137 Research-Irradiator “Mark-I Model M68A” (J. L. Shepherd & Associates, San Fernando, CA 91340). The unit is comprised of a sealed pencil-like (37 cm long) radioactive Cesium-137 source producing 662 keV gamma (γ) radiation. *Ldlr*^−/−^ mice were placed in a cylindrical container (inner diameter 20.2 cm, height 13.4 cm). Thin black plastic strips were arranged to create wedge-shaped partitions for 8 mice per cylinder. A round top-cover of clear plastic with a wedge-shaped cut-out was used to conveniently place/retrieve mice one at a time. The mice-loaded container was placed on an acrylic stand 30.1 cm above the axis of the Cesium source. A dose of 2 Gy was delivered in this geometry. Dose uniformity was within 5%. The in-air dose-rate at the irradiation height was measured with an ion-chamber employing the Task-Group Report 61 of the AAPM (American Association of Physicists in Medicine). The chamber had been calibrated by M. D. Anderson’s ADCL (Accredited Dosimetry Calibration Laboratory). The chamber calibration is traceable to NIST (National Institute of Standards and Technology).

### Serum Lipid Profile Analysis

Mice fasted overnight were euthanized with CO_2_, and whole blood was collected in a 1.5 ml tube. Whole blood was allowed to clot for 45 min at RT and centrifuged at 1,500 × g for 30 min at 4°C. The levels of cholesterol (high- and low-density lipoprotein, HDL and LDL, respectively) were determined using cholesterol assay kits (Cat#EHDL-100, Bioassay System, USA).

### Quantification of Total and Phosphorylated p90RSK After PCL

For total p90RSK and phosphorylated p90RSK density analysis after PCL, nonsaturated images from the LCA of IR and non-IR treated groups were analyzed with ImageJ software to quantify integrated density of total and phosphorylated p90RSK staining per relevant area (intima vs. media) within each image.

### Statistical Analysis

Data are presented as means ±  SEM. Differences between two independent groups were determined using the Student *t*-test (two-tailed) and, when applicable, one-way ANOVA followed by Bonferroni post hoc testing for multiple group comparisons using the Prism software program (GraphPad Software, La Jolla, CA, USA). When groups exhibited unequal variances, Welch’s ANOVA was applied to perform multiple group comparisons. *p* values less than 0.05 were considered statistically significant and indicated by an asterisk in the figures.

## Results

### Effect of Doxorubicin (Dox) on D-Flow-Induced Plaque Formation After PCL

**Figure 1:**
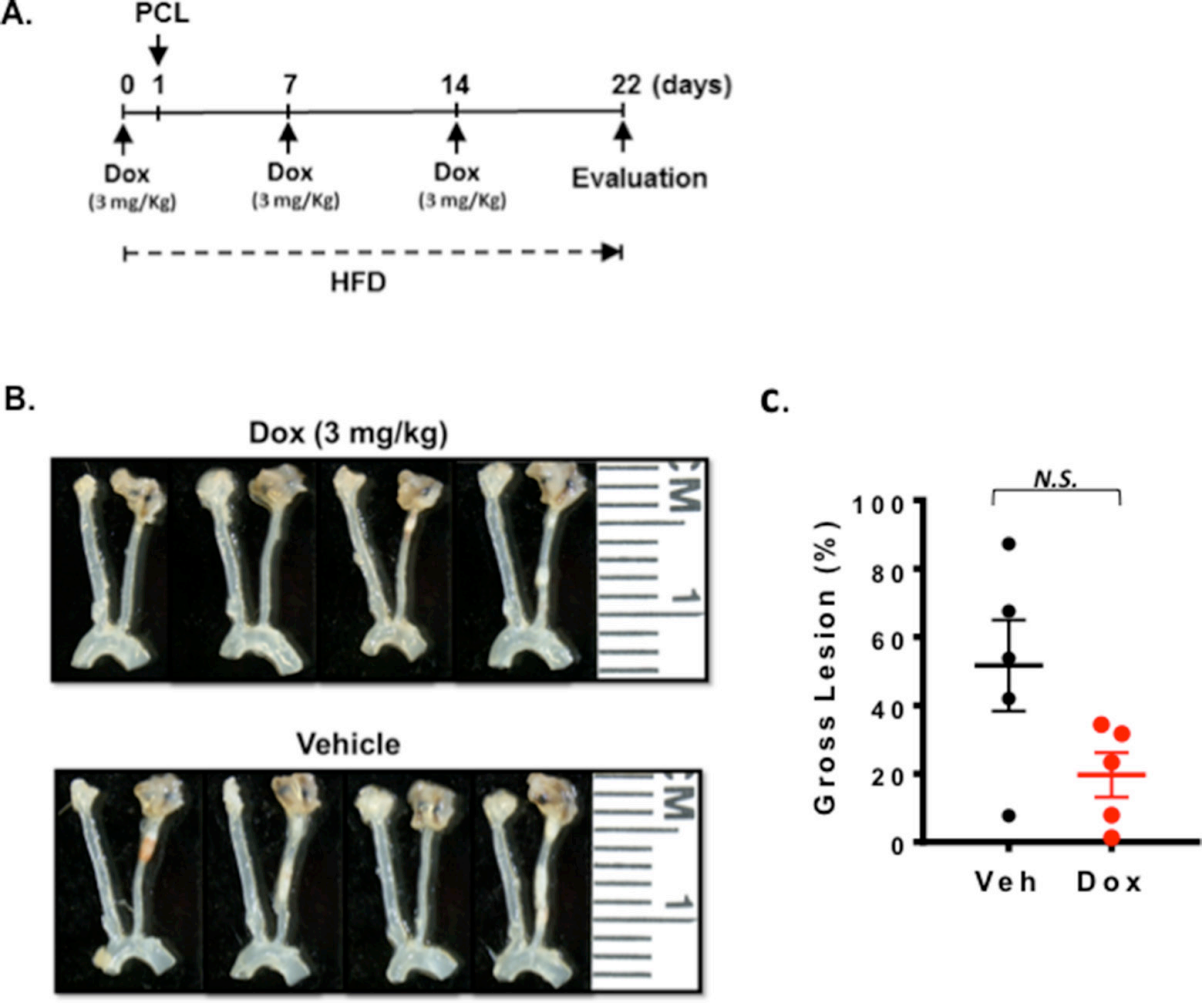
Doxorubicin effect on plaque formation after PCL. All the animals did not show any clear health problem before each experiment. **(A)** The scheme of the study design of doxorubicin treatment and PCL. **(B)** Representative gross lesions from doxorubicin- or saline-treated groups after 3 weeks of PCL in *Ldlr*^−/−^ mice. Left carotid artery is the right-side vessel (smaller of the two carotids) in each sample. **(C)** Gross lesion size after 3 weeks of PCL surgery (% lesion area of total LCA area). 5 males in doxorubicin-treated group and 3 males and 2 female in a vehicle group.

As we presented in the introduction, Dox may induce vascular injury (30, 31). To test this in mice, we injected 3 mg/kg of Dox before and after PCL and harvested carotids 3 weeks later (Figure 1A). As shown in Figure 1B, C, we found that Dox treatment did not accelerate d-flow-induced plaque formation compared to saline treated mice with this study design.

### Cisplatin Injection Before 5 Weeks of PCL Did Not Accelerate Plaque Formation

**Figure 2:**
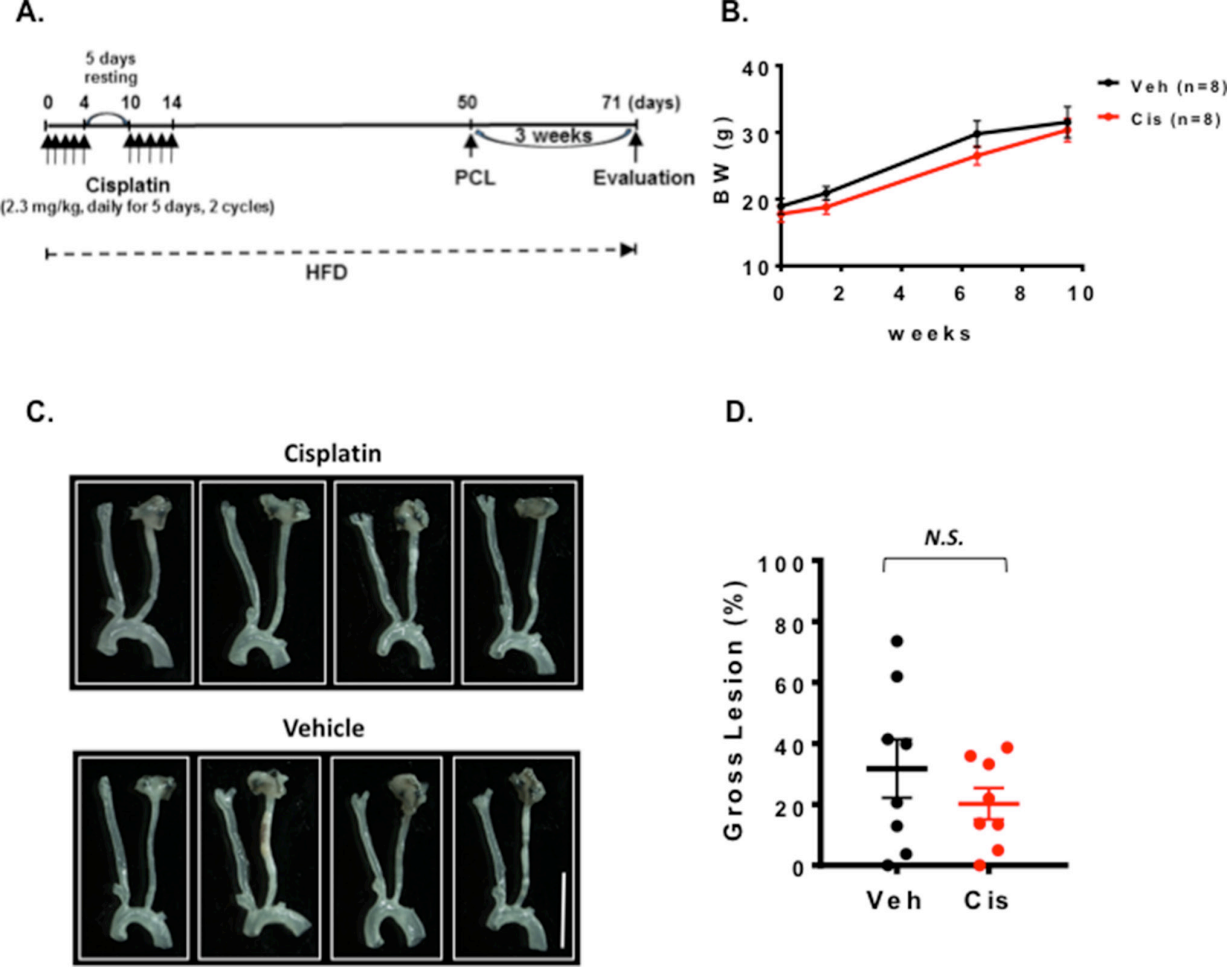
Cisplatin treatment did not change the plaque size after PCL. All the animals did not show any clear health problem before each experiment. **(A)** The scheme of the study design of cisplatin treatment and PCL. (B) Changes in body weight after saline, cisplatin, and PCL surgery (mean ± SEM). **(C)** Representative gross lesions from cisplatin- and saline-treated groups after 3 weeks of PCL in *Ldlr*^−/−^ mice. Scale bar, 0.5 cm. **(D)** Gross lesion size after 3 weeks of PCL surgery (% lesion area of total LCA area). 5 males and 3 females in both cisplatin and vehicle-treated groups.

The role of cisplatin in promoting vascular injury has been reported (32). We first treated mice with 2.3 mg/kg cisplatin via 2 cycles of daily intra-peritoneal injections for 5 days with 5 days of rest between the two cycles. We waited for 5 weeks until the mouse condition became stable after the chemotherapy, and then performed PCL (Figure 2A). We harvested carotids and studied plaque sizes. We found that cisplatin treatment showed no effect on body weight (Figure 2B) and plaque formation after PCL under this experimental condition (Figure 2C,D).

### Plaque Formation Was Accelerated by IR After PCL

**Figure 3:**
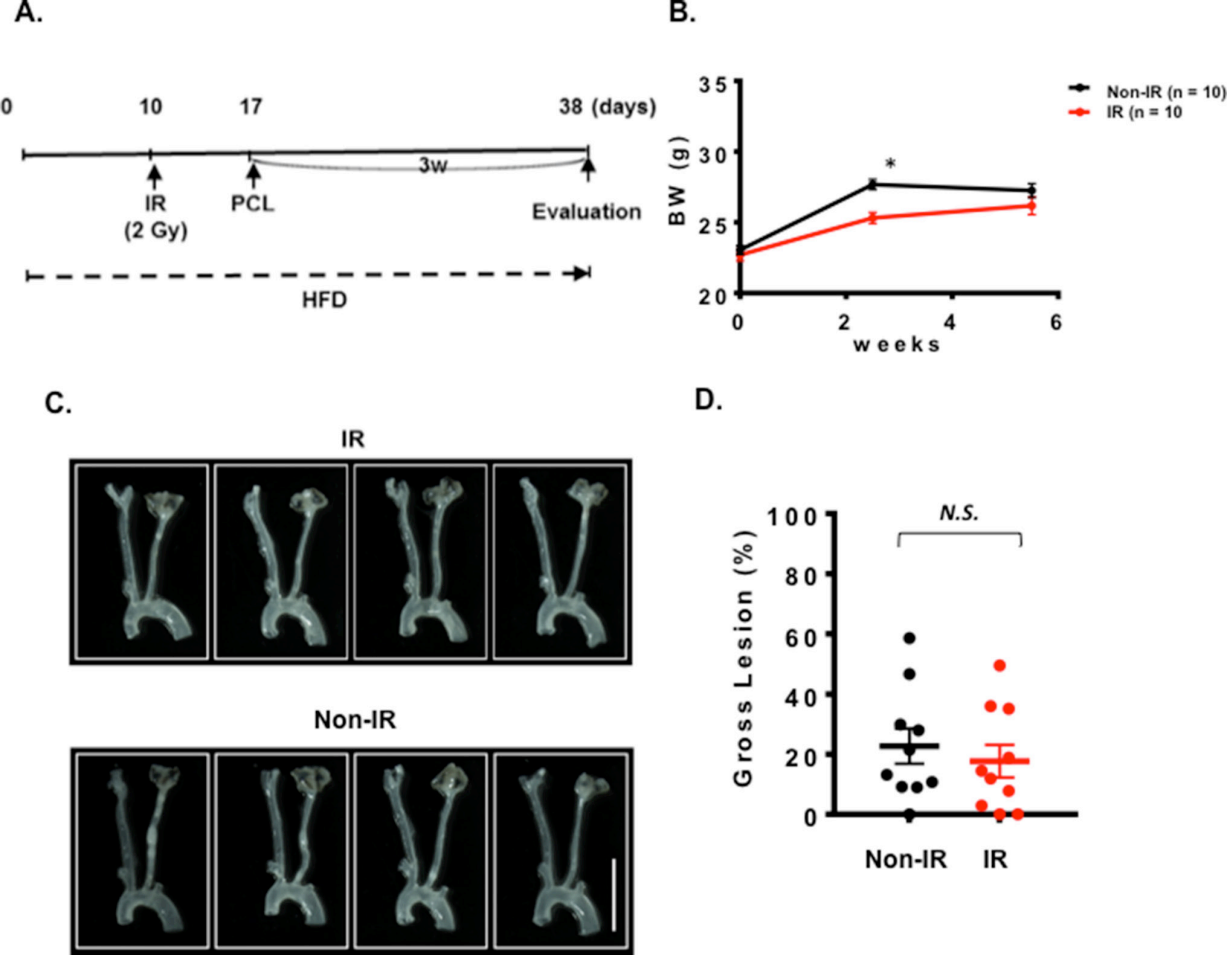
One week interval between IR treatment and PCL did not change the plaque size after PCL. All the animals did not show any clear health problem before each experiment. **(A)** The scheme of the study design of IR treatment and PCL. **(B)** Changes in the body weight after IR, or non-IR treated group and PCL surgery. (mean ± SEM) **(C)** Representative gross lesions from IR- or non-IR-treated groups after 3 weeks of PCL in *Ldlr*^−/−^ mice. Scale bar, 0.5 cm. **(D)** Gross lesion size after 3 weeks of PCL surgery (% lesion area of total LCA area). 10 males in IR-treated group and 10 males on control non-IR group.

**Figure 4:**
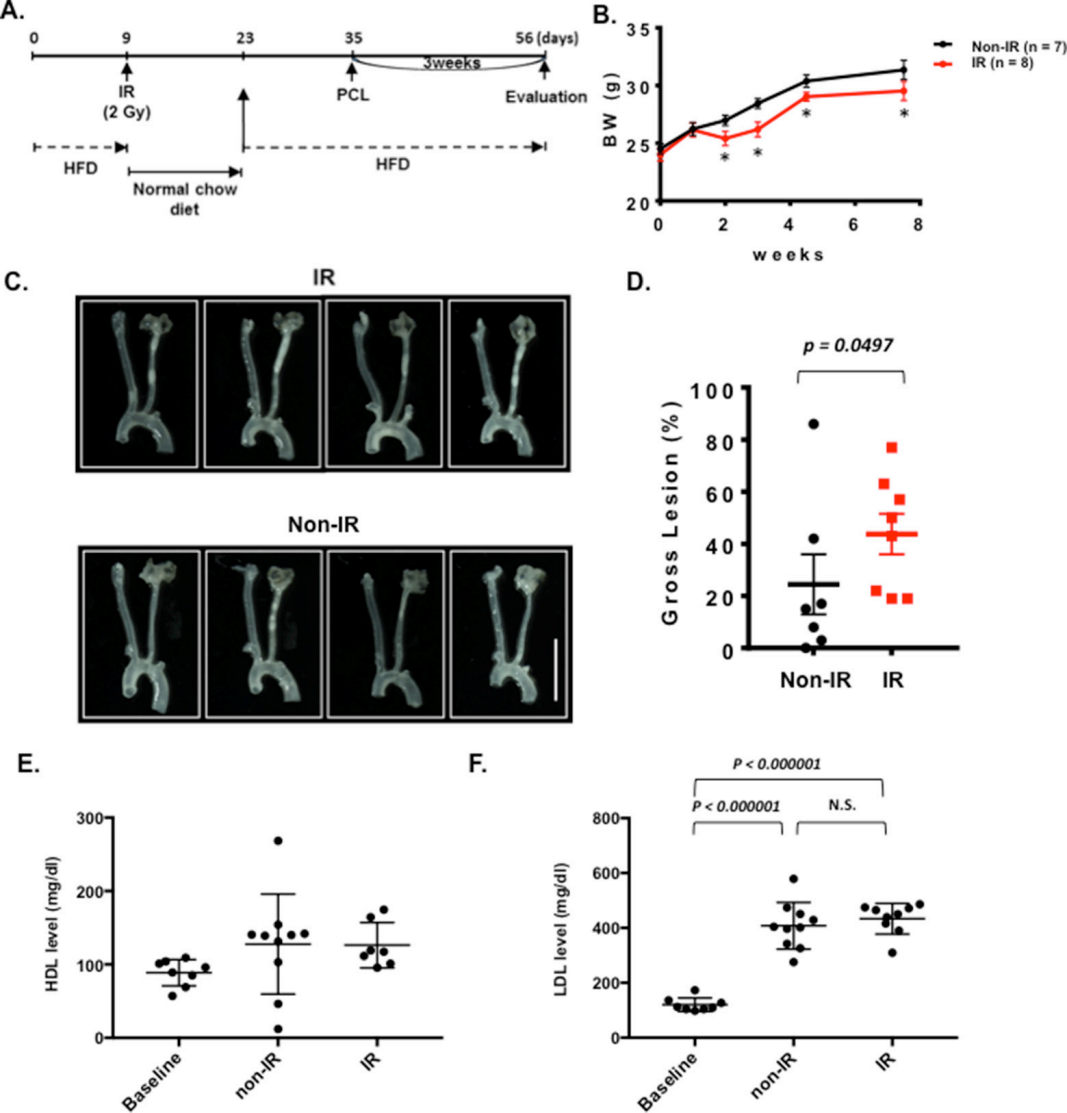
Interval of 26 days between IR treatment and PCL accelerates plaque development after PCL. All the animals did not show any clear health problem before each experiment. **(A)** The scheme of the study design of IR treatment and PCL. **(B)** Changes in body weight in the IR and non-IR treatment groups. (mean ± SEM) **(C)** Representative gross lesions from the IR and non-IR treatment groups 3 weeks after PCL in *Ldlr*^−/−^ mice. Scale bar, 0.5 cm. **(D)** Gross lesion size after 3 weeks of PCL surgery (% lesion area of total LCA area). **(E-F)** HDL and LDL levels in IR and non-IR treatment groups. 8 males in IR-treated group and 7 males in control group.

We could not detect accelerated plaque formation by doxorubicin and cisplatin in our PCL model under the conditions we studied. Mancuso et al. have reported that atherosclerotic lesion formation in irradiated athero-prone ApoE^−/−^ mice was accelerated, especially in the d-flow area (33). Therefore, we applied 2 Gy of γ radiation and performed PCL surgery 1 week later. We harvested carotids 3 weeks after PCL (Figure 3A). As shown in Figure 3B, we found a significant decrease in body weight after IR compared to the non-IR group. We found no difference in d-flow-induced plaque formation between IR and control groups (Figure 3C,D). Another group of mice was allowed to regain body weight after IR, and PCL surgery was performed subsequently (Figure 4A). In this regimen, mice were fed a high fat diet for 9 days, received 2 Gy of IR, and fed a normal chow diet for 14 days during which the body weight of the irradiated mice recovered. The normal diet was given during the recovery period in order to avoid the extra plaque formation induced by high fat diet in both IR and non-IR mice. High fat diet feeding was resumed when the body weight returned to the level of the non-irradiated, age matched normal mice, and PCL surgery was performed 12 days later. Carotids were harvested 3 weeks afterwards for atherosclerotic plaque formation (Figure 4A,B). With this study design, we were able to detect IR-induced enhancement of plaque formation after PCL surgery at the gross anatomical level (Figure 4C,D). We found significant increase of LDL levels in both non-IR and IRgroups after HFD and surgery, but we did not observe any differences in HDL and LDL levels between IR and non-IR groups (Figure 4E,F). These lipid data after surgery (non-IR and IR groups) were obtained at the time of sacrifice. The relatively large variation of HDL levels in the non-IR group may be due to the combination of the surgery and normal chow diet feeding for two weeks. IR may decrease this procedure-related variation.

**Figure 5:**
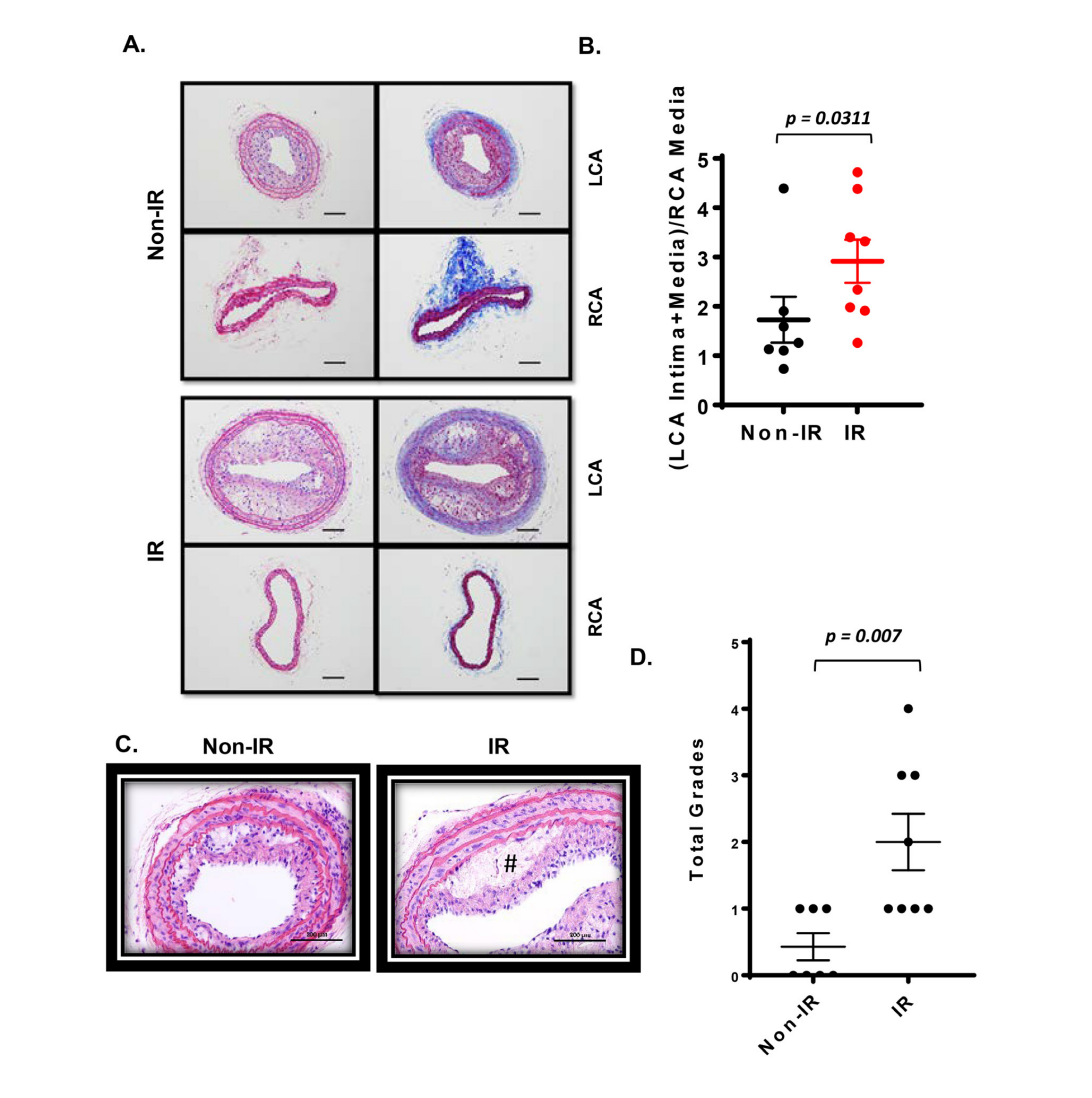
Interval of 26 days between IR treatment and PCL enhanced the plaque size and vulnerable plaque formation detected in cross-sections. All the animals did not show any clear health problem before each experiment. **(A)** Atherosclerotic lesions 3 weeks after PCL in the left carotids isolated from the IR and non-IR treatment groups are shown. The unligated right carotid artery (RCA) from the same mouse is used as control (shown in lower panels of IR and control). Representative images are shown after H&E (left) and Masson trichrome (right) staining. Scale bars, 200 µm. **(B)** The intimal lesion area and the media area were determined in cross-sections of both ligated LCAs and control RCAs, and the ratio of (LCA intima + media)/RCA media was calculated. Data represent mean ± SEM. *p < 0.05. **(C)** Representative images of the necrotic core from the IR and non-IR treatment groups are shown after H&E staining. # denotes necrotic core. **(D)** Necrotic core formation was quantified by a grading system as described in the methods. 8 males in IR-treated group and 7 males in control group.

**Figure 6:**
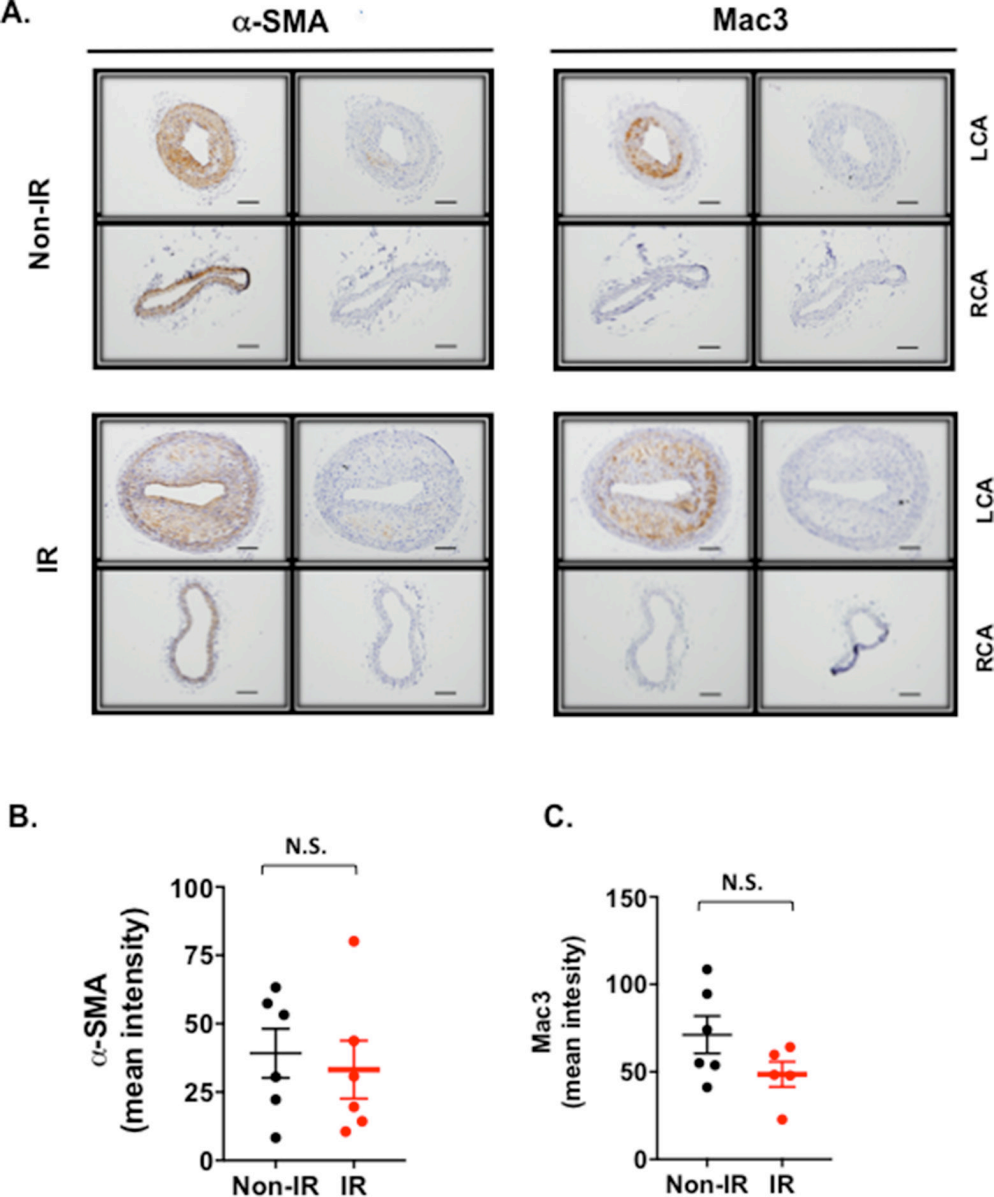
Vascular smooth muscle cell and macrophage contents in lesions after partial carotid ligation. **(A)** LCA and RCA sections obtained from d-flow-induced atherosclerotic areas were immunostained with Mac3 (macrophages) or α-SMA (smooth muscle cells) antibodies. Representative immuno-histochemical images show macrophage infiltration into the intimal lesions in LCA but not in RCA. They also show that macrophage infiltration is accelerated in IR-treated mice. Scale bars, 200 µm. **(B, C)** The mean pixel intensities of α-SMA **(B)** and Mac3 **(C)** staining signals in the intima layer were measured as described in methods. Data represent mean ± SEM. 5 males in IR-treated group and 5 males in control group.

Plaques formed under these conditions were characterized using routine histological methods. Robust plaques were seen in the irradiated and partially ligated carotid artery compared to the non-IR counterpart (Figure 5A,B). Interestingly, we found a clear necrotic core (non-cellular area) formation in the plaques of the IR-treated group, but less in the non-IR group (Figure 5C,D). In plaques of both groups, we detected a significant increase in macrophage infiltration into the plaque, which distinguishes these plaques from the restenotic lesions seen during vascular intimal formation after vascular injury (Figure 6). We did not observe a significant difference in the anti-α-SMA-positive cells in the intimal layer between control and IR groups (Figure 6). These data suggest that IR treatment induces not only larger plaques but also a more advanced unstable type of plaques characterized by the increased necrotic cores.

### IR Increased p90RSK Activation in Vivo

**Figure 7:**
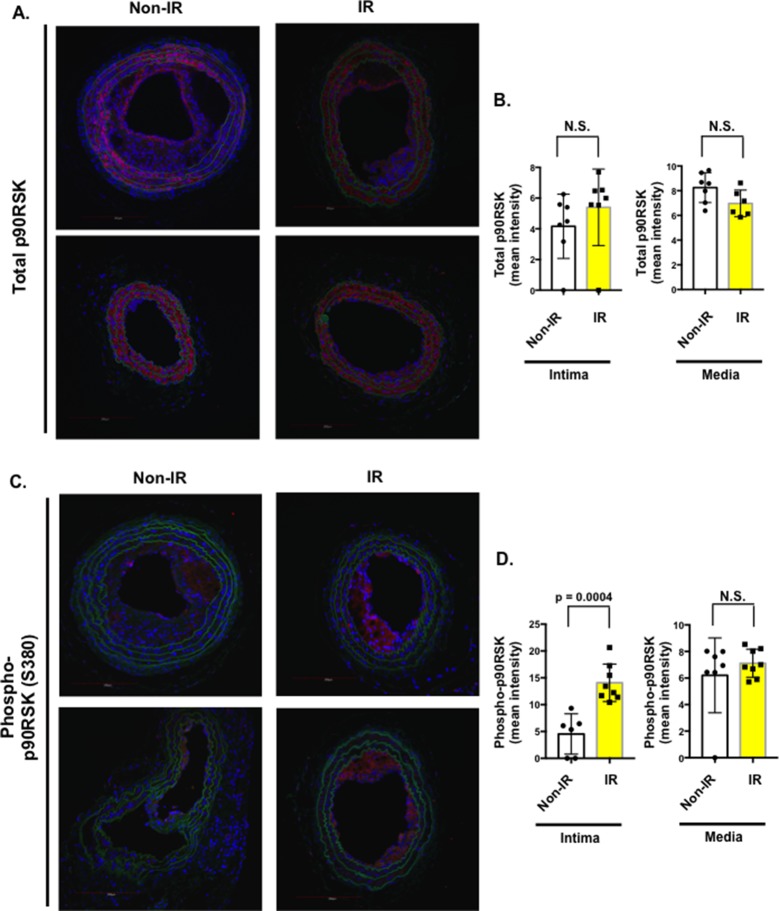
Interval of 26 days between IR treatment and PCL accelerated both phosphorylation and expression of p90RSK after PCL. All the animals did not show any clear health problem before each experiment. **(A, C)** LCA sections after PCL were immuno-stained with anti-total-p90RSK **(A)** or anti-phopho-p90RSK antibody **(C)**. Two representative images for total-p90RSK **(A)** and anti-phopho-p90RSK **(C)** in atherosclerotic lesions are shown. Note that the level of phosphorylation but not expression of p90RSK was increased in IR-treated mice compared to non-IR group. Scale bars, 200 µm. **(B, D)** Quantification of the mean pixel intensity of total p90RSK **(B)** and phosphorylated p90RSK **(D)** expression in the intima and media after PCL in non-IR and IR groups. Mean ± SD, *p < 0.05.

Previously, we have reported the crucial role of p90RSK activation in atherosclerotic plaque formation (24). However, it remains unclear whether p90RSK is activated in the plaques after IR. As shown in Figure 7, we found a significant increase of phosphorylated p90RSK in the plaque intima after IR treatment compared to those from non-IR group. We did not find any difference in the total p90RSK expression between IR and non-IR groups. These data suggest the possible role of p90RSK activation in IR-mediated enhancement of plaque formation.

## Discussion

It is clear that cancer survivors suffer from higher incidence of cardiovascular events than individuals in the general population. Therefore, it is crucial to establish appropriate animal models that recapitulate this phenomenon for studying the molecular mechanisms and developing therapeutics for the cancer treatment-induced cardiovascular malady. It is well known that disturbed flow accelerates the process of EC inflammation and subsequent atherosclerosis (AS). However, the relationship between disturbed flow and cancer treatment in regulating the development of AS remains unclear. In this study we found that IR (2 Gy) treatment enhanced the size of atherosclerotic plaques after PCL. We also found that IR significantly accelerated formation of vulnerable plaques with the characteristic necrotic core formation. These data suggest that *Ldlr*^−/−^ mouse treated by IR can be a good model for studying AS in cancer survivors.

The IR-enhanced plaque formation in conventional ApoE^−/−^ mice has already been reported (33, 34). But the study reported by Mancuso et al lasted almost 1 year to see enhanced atherosclerosis lesion formation by IR (33). This is not an easy and “affordable” method, and significantly limited the research in cardio-oncology. In addition, the other study did not show any lesion size difference with or without radiation (34). Therefore, previous reported were in disagreement in terms of the size of atherosclerotic plaques. In this report, we have described an affordable and reproducible experimental method to study the effect of cancer therapy on atherosclerotic lesion formation. In addition, atherosclerosis lesion formation at the aortic root and aortic arch areas may not be directly related to disturbed flow-induced atherogenesis, because it has been reported that fibronection expression is higher in these areas (35). By focusing on the lesions formed after PCL, we can evaluate the role of disturbed flow in cancer therapy-mediated vascular dysfunction.

We also tested Dox and cisplatin in our model, but we could not detect accelerated AS with the protocol tested in this study. The crucial role of macrophage proliferation during the process of plaque formation has been well established. Since we treated mice with Dox after PCL surgery, it is possible that Dox treatment inhibited macrophage proliferation in the plaque and inhibited its size. To detect the long term effects of Dox treatment on AS, we may need to add a certain length of waiting period between the end of Dox treatment and PCL surgery. As for the cisplatin treatment, we could not detect any enhancement effect on plaque formation after PCL, even after waiting for 5 weeks after cisplatin treatment to perform PCL surgery. It is well known that cisplatin can cause renal dysfunction. Although we did not examine renal function in this study, it is possible that cisplatin-induced renal dysfunction affected the extent of plaque formation after PCL. These data provide a lesson that it is critical to control non-cardiovascular toxicity of drugs when investigating cardiovascular toxicity of cancer treatments. We could not observe statistically significant differences in the development of atherosclerosis between control and chemotherapy (Dox and cisplatin)-treated groups during our relatively short study periods. It is possible that we may need to extend our study to a much longer time course in order to detect increased atherosclerotic plaque formation by these anti-cancer drugs. To study the late effects of chemotherapy reagents, it will be crucial to minimize the non-cardiovascular acute side effects induced by the chemotherapy reagents and more careful optimization will be necessary.

If we increase the number of animals, we will most likely detect a difference between control and Dox treatment regarding lesion size. Gross lesion (%) SD was much bigger than cross-section size analysis, which we have performed before. Therefore, based on the current data the optimal number of animals needed to attain statistical significance of *p* < 0.05 with a 90% probability is 22 for each group. But this result will show that atherosclerosis will be down-regulated by Dox in this mouse model. We would like to emphasize here that the goals of this study are: (1) to generate an affordable, reliable, and reproducible mouse model that recapitulates the cancer therapy-induced cardiovascular events in cancer survivors and (2) to generate a mouse model to investigate the interplay between disturbed flow and various cancer therapy in the process of atherosclerotic plaque formation. Therefore, even if we increased the mouse number to 22 for each group, we would only confirm the data based on the *n* = 5 (for each group) studies and this would not lead us to generate a mouse model that recapitulate the cancer therapy-induced cardiovascular events in cancer survivors. Although it is unlikely, we might be able to show that Dox treatment could enhance lesions if we increased the number of animals. For such experiments, we would need more than 28 animals for each group to get the results counter to the data obtained from *n* = 5. This is against our goal to obtain “an affordable, reliable, and reproducible mouse model.” Again, based on our results we could not conclude that Dox was unable to enhance atherosclerotic lesions compared to vehicle control in mice. However, it seems fair to state that increasing the animal number by more than 5 times does not satisfy our goal to create an affordable, reliable, and reproducible mouse model for studying mechanisms of cardio-oncology.

When we treated mice with IR and waited for a week to perform PCL surgery, we were unable to find any difference in the plaque size between non-IR and IR groups. As shown in Figure 3B, we saw a rapid decrease in body weight after IR. It is possible that this is due to lost appetite or reduced nutrient absorption in the intestine after IR, and this may affect the development of plaque formation. In a different group, we waited for 26 days for the body weight to recover and then performed PCL surgery. In such mice, we detected significantly enhanced IR-induced plaque formation. In order to develop a cancer therapy-induced AS model, it may be important to maintain the body weight after cancer therapy interventions for enhancing the development of plaque formation.

In this study we also found increased phosphorylation of p90RSK in the plaque. Previously, we have reported a crucial role of p90RSK activation in plaque formation and that IR can increase p90RSK activation in both endothelial cells and macrophages (data not shown). Therefore, it is possible that the increase in p90RSK activation in the plaque after IR contributes to accelerated development of AS. Future study will be necessary to clarify this issue.

There are several limitations in this study. For example, the role of p90RSK activation in radiation-mediated plaque formation remains unclear. However, we have already reported that p90RSK can phosphorylates ERK5, which has a significant role in atherosclerotic plaque formation (24). Since we found in this study that p90RSK activation was significantly increased in the plaque, to determine the biological consequence of the increased p90RSK in this model, it will be necessary to generate macrophage- or vascular smooth muscle cell-specific triple p90RSK 1, 2, 3 knock out mice and to determine the total p90RSK null effect on the plaque formation. However, this will be beyond the scope of this study, and we will perform these experiments in our future studies. Since the purpose of this study is to develop and provide a mouse model to the research community for studying the cancer therapy-induced cardiovascular disease and its relationship to disturbed flow, we think that studying the role of p90RSK in this model is not critical at this time. Because, at present, there is no mouse model for studying cardio-oncology, we feel that there is a strong need to develop an animal model of the cancer therapy-induced cardiovascular toxicity.

In this paper, we included certain negative data. These data provide the research community what kind of issues one needs to be aware of for establishing an animal model for studying cardio-oncology. The negative data (not increased atherosclerosis formation after partial carotid ligation) by using Dox and cisplatin are not to conclude that they cannot increase plaque formation, but rather to state that under the conditions we tested, we could not detect increased plaque formation after the Dox and cisplatin treatment. It is still possible that under different experimental conditions, these drugs have athero-enhancing effects, and these issues can be investigated in future studies by us or other investigators. It is hoped that our negative data can help other investigators to save their time, and give some ideas how to optimize the conditions of cancer treatment regimens to establish animal models for studying cardio-oncology. These issues are also supported by the ARRIVE guideline (36), because ARRIVE guideline clearly states that animal models should have translational aspect to human biology, otherwise they strictly prohibit to do animal studies which do not recapitulate human illnesses. Animal studies are necessary which phenocopy human pathology and this cannot be done using non-animal experiments such as cell systems. We did not waste animals to perform experiments which are not relevant to human biology and cardio-oncology research. For example, we did not pursue the Dox effect on inhibiting plaque formation (which was opposite to the human pathology), in accordance of the policy of ARRIVE guide line (36). Lastly, showing negative data will provide the idea how to reduce the future use of animals.

In summary, we have developed a susceptible mouse model, using partial carotid ligation protocols, for the study of disturbed flow effects on atherosclerosis in cancer treatments. This protocol can be used to investigate the effects of IR on d-flow-induced atherosclerosis. In our future work, the contribution of endothelial, vascular smooth muscle cells and macrophage p90RSK activation on IR-induced enhancement of atherosclerotic lesion will be investigated with this model.

## Ethics Statement

Experimental procedures were approved by the Institutional Animal Care and Use Committee (IACUC) at Texas A&M Institute of Biosciences and Technology at the University of Texas MD Anderson Cancer Center.

## Author Contributions

KK performed experiments, interpreted data and wrote the manuscript. YW, SKo, YF, HV, BV, TT, JM, and YG performed experiments and interpreted data. JG-A, ZP, SM, SKr, and MH interpreted data. KF and JA interpreted data and wrote the manuscript.

## Conflict of Interest Statement

ZP is employed by  KBRwyle. The other authors declare that the research was conducted in the absence of any commercial or financial relationships that could be construed as a potential conflict of interest.
