# Supplementary figures and images for: Developing a Reliable Mouse Model for Cancer Therapy-Induced Cardiovascular Toxicity in Cancer Patients and Survivors
Source: Front Cardiovasc Med. 2018 Apr 5;5:26. doi: 10.3389/fcvm.2018.00026 (PMC5896304; doi:10.3389/fcvm.2018.00026)

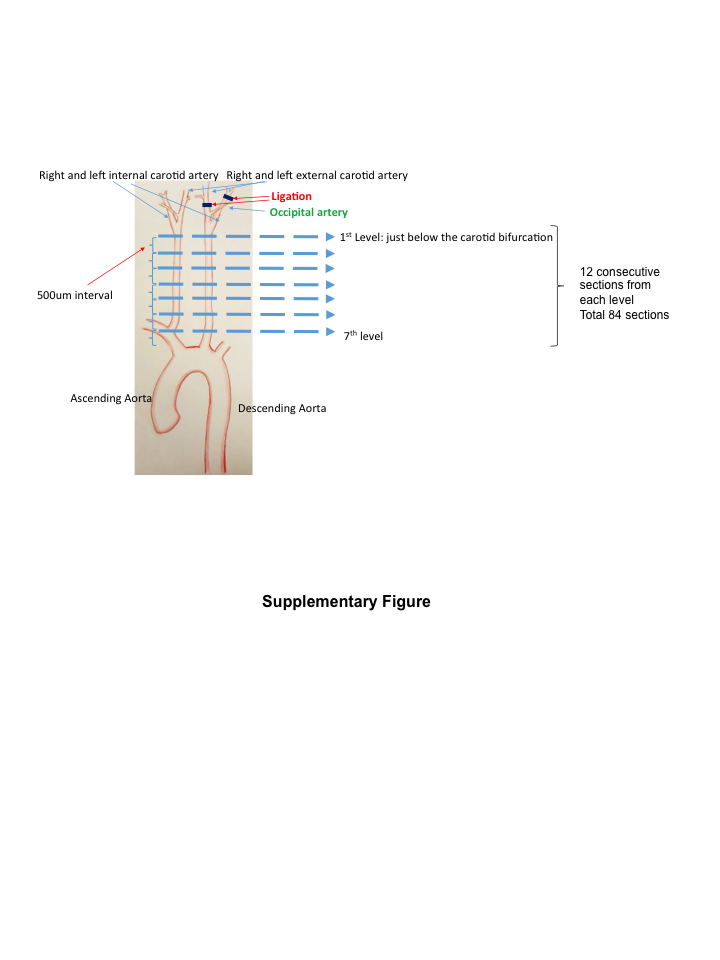

Supplement: Supplementary file 1 [file Image1.tif]
